# Diurnal Harvest Cycle and Sap Composition Affect Under-Skin Browning in ‘Honey Gold’ Mango Fruit

**DOI:** 10.3389/fpls.2019.01093

**Published:** 2019-09-13

**Authors:** Anh T. San, Peter J. Hofman, Daryl C. Joyce, Andrew J. Macnish, Jose R. Marques, Richard I. Webb, Guoqin Li, Heather E. Smyth

**Affiliations:** ^1^Sub-institute of Agriculture Engineering and Postharvest Technology, Ho Chi Minh City, Vietnam; ^2^Maroochy Research Facility, Department of Agriculture and Fisheries, Nambour, QLD, Australia; ^3^School of Agriculture and Food Sciences, The University of Queensland, Gatton, QLD, Australia; ^4^Ecosciences Precinct, Department of Agriculture and Fisheries, Brisbane, QLD, Australia; ^5^Centre for Microscopy and Microanalysis, The University of Queensland, St Lucia, QLD, Australia; ^6^School of Food Science, Shaanxi Normal University, Linfen, China; ^7^Queensland Alliance for Agriculture and Food Innovation, The University of Queensland, Coopers Plains, QLD, Australia

**Keywords:** mango, sap, under-skin browning, volatiles, diurnal harvest cycle

## Abstract

Under-skin browning (USB) is an unsightly physiological disorder that afflicts ‘Honey Gold’ mango fruit. Under-skin browning symptoms develop after harvest upon the interaction of physical abrasion and physiological chilling stresses. Less understood preharvest and/or harvest factors may also influence fruit susceptibility to USB. In this study, we examined the impact of harvest time during the diurnal cycle and fruit sap components on USB development. Fruits were harvested at 4- to 6-h intervals, lightly abraded with sandpaper to simulate vibration damage during refrigerated road transport, held at 12 ± 1°C for 6 days, transported to the research facilities and ripened before USB assessment. Spurt and ooze sap from the fruit were collected at each harvest time. The samples were separated and analysed by gas chromatography–mass spectrometry. Fruit harvested at 10:00, 14:00 and 18:00 h had 3- to 5-fold higher incidence of USB than did those picked at 22:00, 2:00 and 6:00 h. Sap concentrations of the key aroma volatile compounds 2-carene, 3-carene, α-terpinene, *p*-cymene, limonene and α-terpinolene were higher for fruit harvested at 14:00 h compared to those picked at other times. In the fruits harvested in the afternoon, abraded skin treated with spurt sap sampled at 14:00 h had 14.3- and 29.0-fold higher incidence and severity, respectively, of induced browning than did those treated with sap collected at 6:00 h. The results showed that fruit harvested in the afternoon were more susceptible to USB than those picked at night or in early morning. The diurnal variation in fruit sensitivity was evidently associated with specific compositional differences in sap phytotoxicity. Topical application to the fruit skin of pure terpinolene and limonene resulted in induced USB damage, whereas pure carene and distilled water did not. Microscopy examination showed that while skin damage caused by pure terpinolene and limonene was not identical to USB per se, similarities suggested that sap components cause USB under inductive commercial conditions. Considered collectively, these findings suggest that night and early morning harvesting will reduce USB and thus improve the postharvest quality of Honey Gold mango fruit.

## Introduction

The ‘Honey Gold’ mango (*Mangifera indica* L.) is an Australian cultivar first commercialised in or around 2002 (Dillon et al., 2013). It has juicy fibre-free flesh and a pleasant flavour when ripe (Marques et al., 2012). However, an unsightly physiological skin disorder termed ‘under-skin browning’ (USB) can cause substantial economic loss in the marketplace due to consequent poor fruit appearance (Hofman et al., 2010b). Under-skin browning is visible beneath the skin as diffuse discoloured areas, but with no damage to the flesh (Marques et al., 2012). The epidermis and cuticle can sometimes also take on an opaque appearance. Among commercial Australian mango cultivars, Honey Gold is the most susceptible to USB (Winston et al., 2014). Physicochemical mechanisms causing USB and its control are largely unknown. General symptoms appear to reflect discolouration of latex vessels and surrounding cells (Marques et al., 2012). Under-skin browning development is typically associated with exposure of fruit to chilling temperatures (i.e. 12–13°C) combined with vibration during prolonged transport (Marques et al., 2012). The use of soft packaging options, such as foam liners that may reduce vibration damage during transport of Honey Gold mango, combined with delayed temperature reduction after harvest has been shown to reduce USB in commercial practice (Marques et al., 2016).

Mango fruit and stems, including the fruit pedicel and peduncle, are characterised by an internal longitudinal multilayered network of branching resin ducts (Joel, 1980). The extensive mango plant laticifer system stores sap and can leak resinous secretions (Joel and Fahn, 1980). While the fruit is connected to its peduncle, the sap or latex in the resin ducts is generally under turgor pressure. When the peduncle is broken and the resin ducts are severed, sap typically spurts and/or oozes out. Mango sap separates naturally into an oily yellow-brown nonaqueous phase and a milky viscous aqueous phase (Loveys et al., 1992). The nonaqueous phase contains mono-terpenes (Saby John et al., 1999) and alk(en)ylresorcinols (Hassan et al., 2009). The aqueous phase has high polyphenol oxidase (PPO) and peroxidase (POD) activities (Saby John et al., 2003).

Contact of the fruit surface with sap exudate can lead to skin injury termed ‘sapburn’ (Loveys et al., 1992). Desapping mangoes using alkaline detergents that deactivate damaging sap components is commercially practiced to reduce sapburn (Johnson and Hofman, 2009). Applying either whole sap or the nonaqueous phase (but not the aqueous phase) to the skins of several Indian cultivars caused sapburn, presumably due to terpenoids in the sap and PPO and POD in the skin (Saby John et al., 2002). The sap terpenoid composition varies considerably among mango cultivars (Loveys et al., 1992; Saby John et al., 1999). Varying terpenoids may be responsible for sapburn in different mango cultivars. Limonene, ocimene, β-myrcene and α-pinene caused sapburn on several Indian mango cultivars (Saby John et al., 2002). Terpinolene was strongly associated with sapburn on ‘Kensington Pride’ fruit (Loveys et al., 1992). Also, differing sapburn incidence has been reported for fruit harvested at different times of the day, presumably due to varying sensitivity of the mango fruit skin (Maqbool et al., 2007; Amin et al., 2008).

We investigated the relationship between diurnal fruit harvest time and sap composition on the propensity of Honey Gold mango fruit to develop USB. The working hypothesis was that Honey Gold mango fruit harvested in the afternoon would have lower turgor and therefore be less firm, and so relatively less susceptible to physical damage. It was also considered that this response may be related to varying sap physicochemical characteristics at different diurnal harvest times. Fruits were harvested at varying times over 24 h, and USB susceptibility and the concentrations of key aroma volatiles in the sap were determined. The anatomy of USB symptoms was also examined. It was proposed that a better understanding of the mechanisms involved in the putative diurnal effect, including any potential differences in sap composition, may provide deeper insight to inform practical measures to minimise USB incidence in commercial consignments.

## Materials and Methods

### Experiment 1: Effect of Diurnal Harvest Cycle on USB Expression and Sap Components

Green-mature (i.e. 0–10% yellow on the skin surface) Honey Gold mango fruit of uniform size were harvested at commercial maturity (16.8 ± 1.0% dry matter) from a commercial orchard near Katherine, Northern Territory, Australia (14.28°S, 132.16°E). Trees were grown under standard commercial practices (Kernot et al., 1999; Winston et al., 2015). Field temperature and relative humidity (RH) during the harvest periods were recorded (Tinytag Ultra 2 logger, Hasting Data Loggers, Australia).

Two fruits from each of 10 trees in four replicate orchard rows (n = 80) were harvested every 4 to 6 h in the first fruiting season (viz., 6:00, 10:00, 14:00, 18:00 and 24:00) and, due to cloudy conditions in the first day, additionally at 14:00 the next day under sunny conditions). In the second consecutive fruiting season, fruits were collected every 4 h (viz. 6:00, 10:00, 14:00, 18:00, 22:00 and 2:00 h). In both seasons, fruits with 2- to 5-cm-long pedicels attached were carefully transported to a nearby packing shed. The pedicel was broken off at the abscission zone, and the fruits were inverted to allow sap to drain on a desapping rack for 4 to 5 min. The sap from 20 fruits per replicate row was collected into glass vials, which were then covered with aluminium foil and closed with an aluminium screw-on cap. Sap samples were kept on dry ice (BOC, Australia) in a foam container. The fruits were transported by car (ca. 22°C) to the airport and then air-freighted with dry ice from Darwin, Northern Territory, to Brisbane, Queensland, within 1.5 days of harvest. They were then transported in an air-conditioned (ca. 22°C) vehicle from the airport to the research facility in Brisbane, approximately 20 km away. The collected sap was then stored at −20°C pending analysis as described below.

After desapping, fruit susceptibility to USB was determined by a standard USB test developed for this cultivar, which was based on previous observations that gentle abrasion with fine sandpaper often results in USB around the abraded area when fruit is held at 12 to 13°C for 4 to 6 days (Marques et al, 2012). This test consisted of abrading the fruit for 2 s at each of four subsample locations around the largest fruit circumference using a small orbital finishing sander (280 W; Ozito Industries Pty Ltd, Australia) at a speed setting of 5 on a scale of 1 to 6 and with 80-grit sandpaper (Trojan, Australia). Approximately 110 g mass-pressure was applied to the sanding disc. All fruits were abraded within 1 h of harvest and packed into standard single-layer cardboard trays with expanded polystyrene inserts. Half the fruits were placed in a cold room at 12 ± 1°C within 2 h of abrasion (‘no delay’). The other half were held at room temperature and placed in the cold room after 1 day (‘1 d delay’) to reflect the range of packing schedules.

All the fruits from Experiment 1 were kept at 12 ± 1°C for a further 5 days to ‘mimic’ low temperature holding before transport. They were then road-freighted under commercial conditions for about 3 days in a standard 20-pallet, 12.2-m-long refrigerated trailer at ∼14°C to Wamuran in Queensland, a distance of ∼3,100 km from the farm. From Wamuran, the fruits were then transported to Brisbane (ca. 70 km) by car as described above, ripened at 20°C and 80 to 90% RH and assessed for USB expression as described below.

### Experiment 2: Effect of Sap Components on USB

To test the hypothesis that USB diurnal responses are at least partly due to sap characteristics, either whole sap or its components (i.e., spurt or ooze sap) were collected into glass vials as described above from additional green-mature Honey Gold fruit harvested in the morning (6:00 h, n = 40) and afternoon (14:00 h, n = 40) in the second season. Spurt sap was collected either at 6:00 or 14:00 h in the first 10 to 15 s after stem removal. Ooze sap was collected from then on for up to 4 to 5 min, as per Experiment 1. After desapping, the standard USB test was applied (as above) at each of two subsample positions around the largest fruit circumference. A 0.1-ml aliquot of either spurt sap, ooze sap, whole sap or distilled water (control) was placed onto the abraded fruit areas using filter paper as described below. Nine treatments each with seven individual fruit replicates were involved (**Table 1**). Both morning sap applied to afternoon-harvested fruit and afternoon sap applied to morning-harvested fruit were collected and stored in a fridge until the subject fruit had been harvested.

**Table 1:** The average severity (0- to 5-point scale) and incidence (%) of SB in Honey Gold mango skin as affected by sap type and by timing of fruit harvest (Experiment 2).

Treatment^§^	Severity score (0–5) of SB symptoms*	Incidence (%) of treated areas with SB^#^
Water to the afternoon harvested fruit (14:00)	0.0 ^a^	0
**Applied to the morning harvested fruit (6:00)**		
Afternoon whole sap	2.7 ^c^	50
Afternoon spurt sap	1.8 ^b^	57
Afternoon ooze sap	0.0 ^a^	0
**Applied to the afternoon harvested fruit (14:00)**		
Morning spurt sap	0.1 ^a^	7
Morning ooze sap	0.0 ^a^	0
Afternoon whole sap	2.2 ^b^	100
Afternoon spurt sap	2.9 ^c^	100
Afternoon ooze sap	0.0 ^a^	0

^§^At each treated area on the fruit, an aliquot of 0.1 mL of either distilled water, whole sap, spurt sap or ooze sap was placed onto two abraded areas of the fruit harvested at either time. The fruits were then held at 12 ± 1°C for 6 days, road-freighted under commercial conditions for 3 days at about 14°C and ripened at 20°C before being assessed.

*Means (n = 14) for severity with the same letters are not significantly different (P ≤ 0.05) by the LSD test. Fruits were assessed at the ripe stage based on a severity rating scale of 0 = nil to 5 = > 25% of the skin surface area affected.

^#^Incidence = number of treated areas on the fruit skin affected by SB in relation to the total number of treated areas per treatment (n = 14). The incidence data were not statistically analysed as there was but a single percentage value per treatment.

To determine the effects of oil fraction ingredients in mango fruit sap, 0.1 ml of pure terpinolene, limonene, 2-carene (Merck, Germany) or distilled water (control) (**Table 2**) was placed onto 1-cm^2^ filter papers, placed then onto abraded areas of seven individual fruit replicates per treatment as harvested at 14:00 h.

**Table 2:** The average severity (0–5 scale) and incidence (%) of I-USB symptoms in Honey Gold mango skin as affected by the sap components terpinoline, 2-carene and limonene (Experiment 2).

Treatment^§^	Severity score (0–5) of IUSB symptoms*	Incidence (%) of treated areas with I-USB^#^
Water	0.0 ^a^	0
Pure 2-carene	0.1 ^a^	7
Pure limonene	4.0 ^b^	100
Pure terpinolene	4.0 ^b^	100

^§^An aliquot of 0.1 ml of either distilled water, terpinolene, limonene or 2-carene was placed onto two abraded areas of the fruit harvested at 14:00 h. The fruits were then held at 12 ± 1°C for 6 days, road-freighted under commercial conditions for 3 days at about 14°C and ripened at 20°C before being assessed.

*Means (n = 14) for severity with the same letters are not significantly different (P ≤ 0.05) by the LSD test.

^#^Incidence = number of treated areas on the fruit skin affected by I-USB in relation to the total number of treated areas per treatment (n = 14). The incidence data were not statistically analysed as there was a single percentage value per treatment.

Filter papers bearing treatments were covered with pieces of aluminium foil (∼35–40 cm^2^) secured in place under plastic tape to reduce vaporisation. The fruits were then held at 12 ± 1°C for 6 days, road-freighted to Brisbane under commercial conditions and ripened as described above for Experiment 1. They were assessed for damage areas around the abraded sites, as described below.

### Severity and Incidence Assessments

Abrasion-induced USB (A-USB) was defined as damage associated with the abrasion test treatment. It was recorded separately to USB that developed away from abrasion sites and that more typically reflects symptoms seen during handling and transport. Sap application onto abraded fruit generally resulted in brown-coloured damage to the surrounding skin and was defined as sap-induced browning (SB). Application to abraded fruit skin of the predominant sap compounds terpinolene, limonene and 2-carene (Experiment 2) also resulted in brown-coloured damage to the surrounding skin, which was defined as induced USB (I-USB).

Incidences of USB, A-USB, SB and/or I-USB within replicates were calculated as the proportion (%) of either fruit for USB, abrasion sites for A-USB or treated sites for SB or I-USB that developed symptoms relative to the total number of fruit, abrasion sites or treated sites, respectively. The incidence and severity of USB lesions not associated with abrasion treatment (i.e. those that developed on other areas of the fruit during transport) were assessed only in the second fruiting season.

Severities of USB, A-USB, SB and I-USB were calculated as the average severity rating of all fruits in each treatment. Their severities on each fruit were rated the scale (Hofman et al., 2010a): 0 = nil, 1 = < 3% (1 cm^2^) of skin surface affected, 2 = ∼3% (1–3 cm^2^), 3 = ∼10% (3–12 cm^2^), 4 = 10 to 25% (12–25 cm²) and 5 = > 25% of skin surface affected. Fruits were assessed at the ripe stage (i.e. ≥90% of the skin colour had changed from green to yellow).

### Determination of Sap Volume and Composition

The nonaqueous and aqueous phases of sap samples from the diurnal trials in the first and the second fruiting seasons were analysed as described by San et al. (2017). The proportional volumes of whole sap, nonaqueous and aqueous sap phases were measured using a graduated cylinder after the phases had separated. The nonaqueous and aqueous phases of sap collected at each harvest time were separated at 3,000 rpm for 10 min using a 5810 R centrifuge (Eppendorf, Germany). Aliquots of either 0.1 g of whole sap or 0.03 g of nonaqueous phase were mixed for 30 s in 10 ml of distilled water using a ball mill MM400 (Retsch GmbH, Germany), diluted 50 times with distilled water and then diluted a further either 50 or 25 times with distilled water for the whole sap and nonaqueous phase, respectively. To ensure accuracy and precision in the quantitative measurement of the targets, the nonaqueous and aqueous phases of sap samples were then analysed for aroma volatile quantification by using stable isotope dilution analysis, in conjunction with headspace (HS) solid-phase microextraction coupled with gas chromatography–mass spectrometry. The concentrations (mg g^−1^) of hexanal, 2-carene, 3-carene, α-terpinene, p-cymene, limonene, α-terpinolene and ethyl octanoate in the mango samples were referenced to authenticated standard curves and calculated from the peak area ratios for the unlabelled and labelled compounds versus the concentration ratio. The biochemical targets were selected because they are typically the volatiles with the highest concentrations and the greatest contributors to aroma of mango fruit and sap, which had been previously established in the literature for three major Australian mango cultivars, including HG (San et al., 2017). The targets were confirmed during method development, in which an initially untargeted analytical approach was used to ensure that the target volatiles were the most important compounds in the samples.

### Anatomy of USB, A-USB, SB and I-USB

Fruit tissue samples (n = 3) both with and without skin browning symptoms were prepared for light microscopy. Under-skin browning, SB and I-USB on Honey Gold fruit were compared and contrasted at the cellular level. Free-hand sections were cut through the excised tissues (Ruzin, 1999). The tissue sections were transferred to a drop of distilled water on a glass microscope slide and covered with a glass cover slip. The hand sections were viewed and photographed with an Olympus BX61 LM equipped with a DP 70 camera (Olympus, Japan).

### Experimental Design and Statistical Analysis

In Experiment 1, a factorial design was used (six harvest times × two delay treatments), with four orchard rows as replicates. At each harvest time, 20 fruits per replicate comprising four subsample abraded areas per fruit were used for A-USB in the diurnal harvest effect (10 fruit per delay treatment per replicate) and for sap volume and composition (20 fruits per replicate). In Experiment 2, a completely randomised design was adopted with either nine or four treatments for the sap type/harvest time trial or the oil fraction components trial, respectively. Seven individual fruit replicates were used comprising two subsample abraded areas per fruit.

Statistical analyses were performed using Genstat 14 (VSN International Ltd., UK). In Experiment 1, USB incidence was analysed using the generalised linear model procedure with a binomial distribution and logit link. The effects of harvest time and delay treatments were included. Back-transformed means (percentages) are presented. In Experiment 2, incidence data were not analysed statistically as there was only one data value (percentage) per treatment. The general analysis of variance model was used to analyse severity data on both experiments, as well as the volume of sap and the proportion of sap phases. The one-way analysis of variance model was used for concentrations of sap components.

Whenever the treatment effect was significant (*P* ≤ 0.05), pairwise comparisons were made using Fisher protected least significant difference (LSD) test. Vertical bars indicating LSD values or different letters for separating treatment means are presented in graphs and tables, respectively.

## Results

### Experiment 1: Effect of Diurnal Harvest Cycle on USB and A-USB Expression and Sap Components

#### USB and A-USB Incidence and Severity

**Figure 1:**
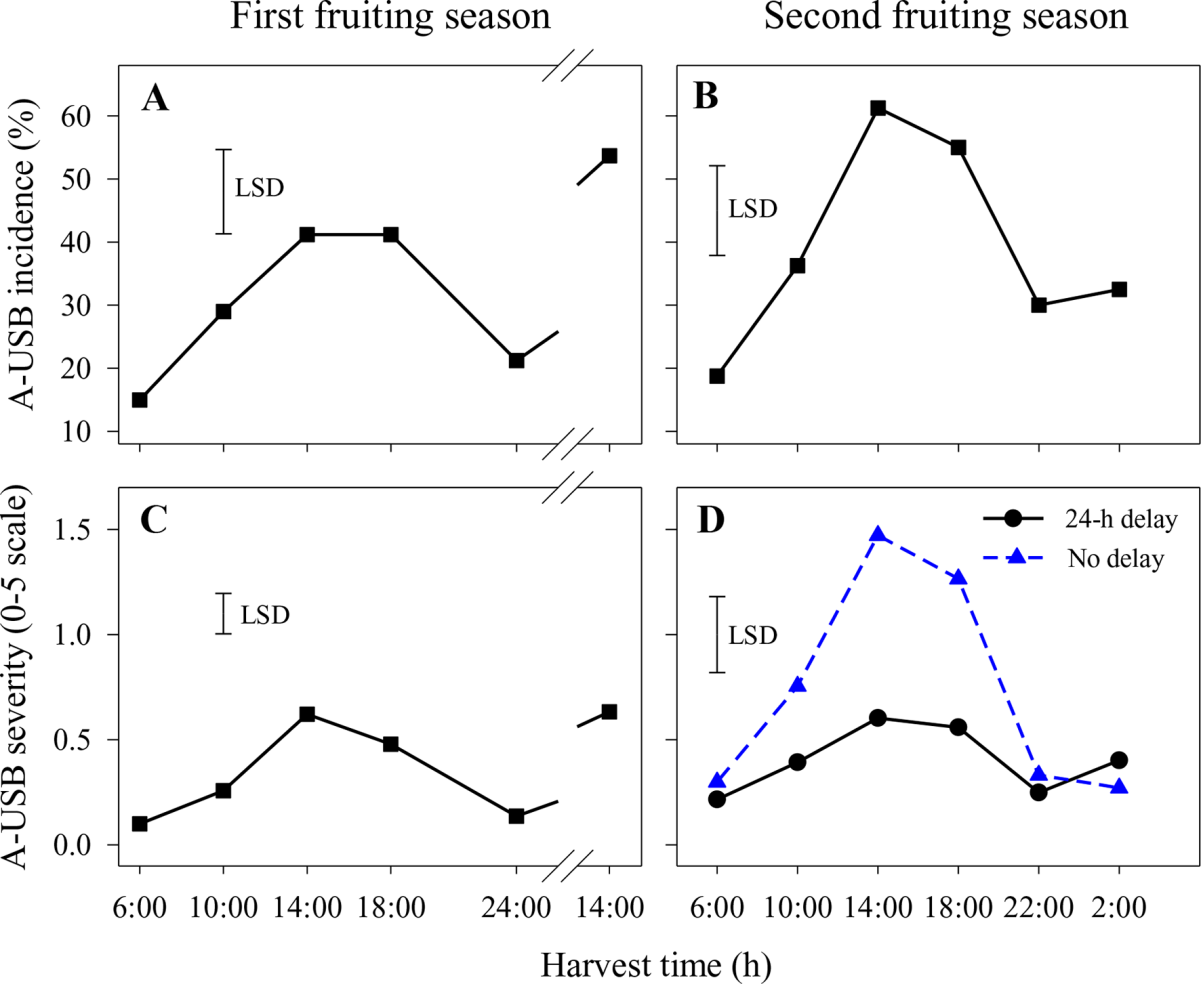
Incidence **(A** and **B)** and severity **(C** and **D)** of under-skin browning that was induced by the abrasion test (A-USB) on Honey Gold mango fruit over the diurnal harvest cycle in the first **(A** and **C)** and the second **(B** and **D)** consecutive fruiting seasons (Experiment 1). Fruits were held at 12 ± 1°C for 6 days either after 24 h of abrasion (‘24-h delay’) or within 2 h of abrasion (‘No delay’), and results were averaged when the differences between these two times were not significant **(A**–**C)**. Fruits were assessed at the ripe stage based on a severity rating scale of 0 = nil to 5 = > 25% of the skin surface area affected. Incidence (%) = number of abraded areas on the fruit skin affected by A-USB in relation to the total number of abraded areas per treatment. The vertical bar on each graph indicates the LSD value (*P* ≤ 0.05) for comparison between harvest time in panels **(A**–**C)** (n = 160) or for the significant interaction (*P* ≤ 0.05) between harvest time and delay treatments in panel **(D)** (n = 80). Break lines on the harvest timescales in graphs A and C indicate a gap of 14 h between assessments.

In the first fruiting season, A-USB incidence and severity were higher in fruit harvested at 14:00 and 18:00 h than in fruit harvested at other times (**Figures 1A, C**). That pattern was repeated for fruit harvested at 14:00 on the second harvest day. There were no significant differences in A-USB severity or incidence between fruit placed in the cold room within 2 h of abrasion and those held for 1 day before cooling. Accordingly, results across both delay treatments were averaged. The interactions between harvest time and delay on A-USB incidence and severity of abraded sites were also not significant.

Likewise, A-USB incidence in the second fruiting season was higher in fruit harvested at 14:00 and 18:00 h compared to 22:00, 2:00 and 6:00 h (**Figure 1B**). There were no significant differences in A-USB incidence between fruit placed in the cold room immediately after abrasion and those held for 1 day before cooling (**Figure 1B**). In contrast, variations in diurnal effects on A-USB severity were more pronounced in fruits placed in the cold room immediately after abrasion than those placed 24 h after abrasion (**Figure 1D**). Considering both seasons, the effects of delay in cooling on A-USB were inconsistent. The interaction between harvest time and delay on A-USB severity of abraded sites was also significant.

**Figure 2:**
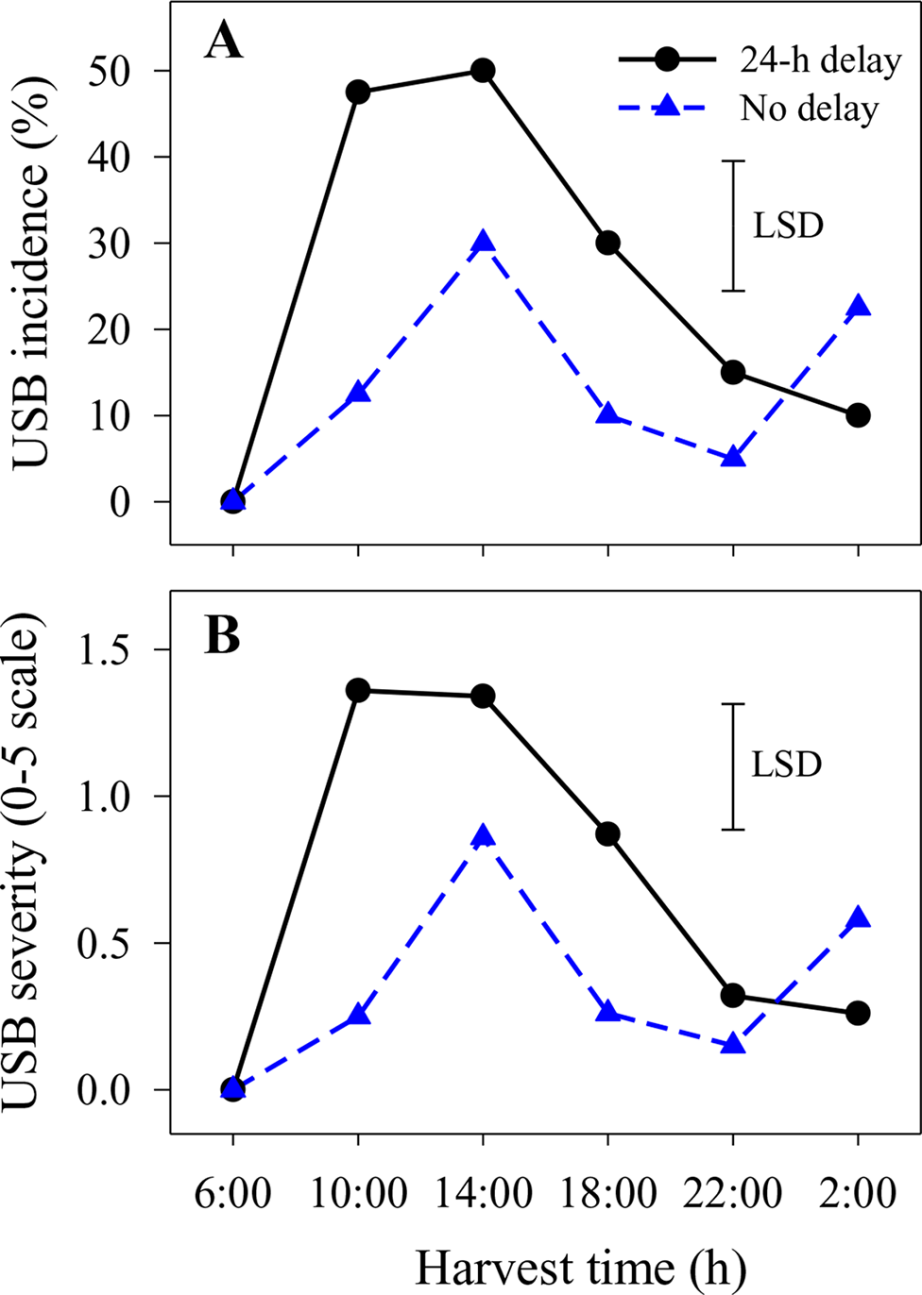
The incidence **(A)** and average severity **(B)** of under-skin browning that was not associated with the abrasion test (USB) on ‘Honey Gold’ mango fruit over the diurnal harvest cycle in the second fruiting season (Experiment 1). Fruits were held at 12 ± 1°C for 6 days either after 24 h of abrasion (‘24-h delay’) or within 2 h of abrasion (‘No delay’). Fruits were assessed at the ripe stage based on a severity rating scale of 0 = nil to 5 = > 25% of the skin surface area affected. Incidence (%) = number of fruit affected by USB in relation to the total number of fruit per treatment. The vertical bar on each graph indicates the LSD value (*P* ≤ 0.05) for the significant interactions between harvest time and delay treatments (n = 20 per data point).

Under-skin browning lesions were also observed on areas of the skin that had not been abraded. In the second season, the incidence and severity of USB not caused by artificial abrasion were higher in fruit harvested at 14:00 h with or without a 1-day delay before cooling or at 10:00 h with a 1-day delay compared with other harvest times (**Figure 2**). Delaying cooling for 1 day resulted in increased incidence and severity in fruit harvested at 10:00, 14:00 and 18:00 h relative to no delay at the same harvest times (**Figure 2**).

#### Sap Composition

**Figure 3:**
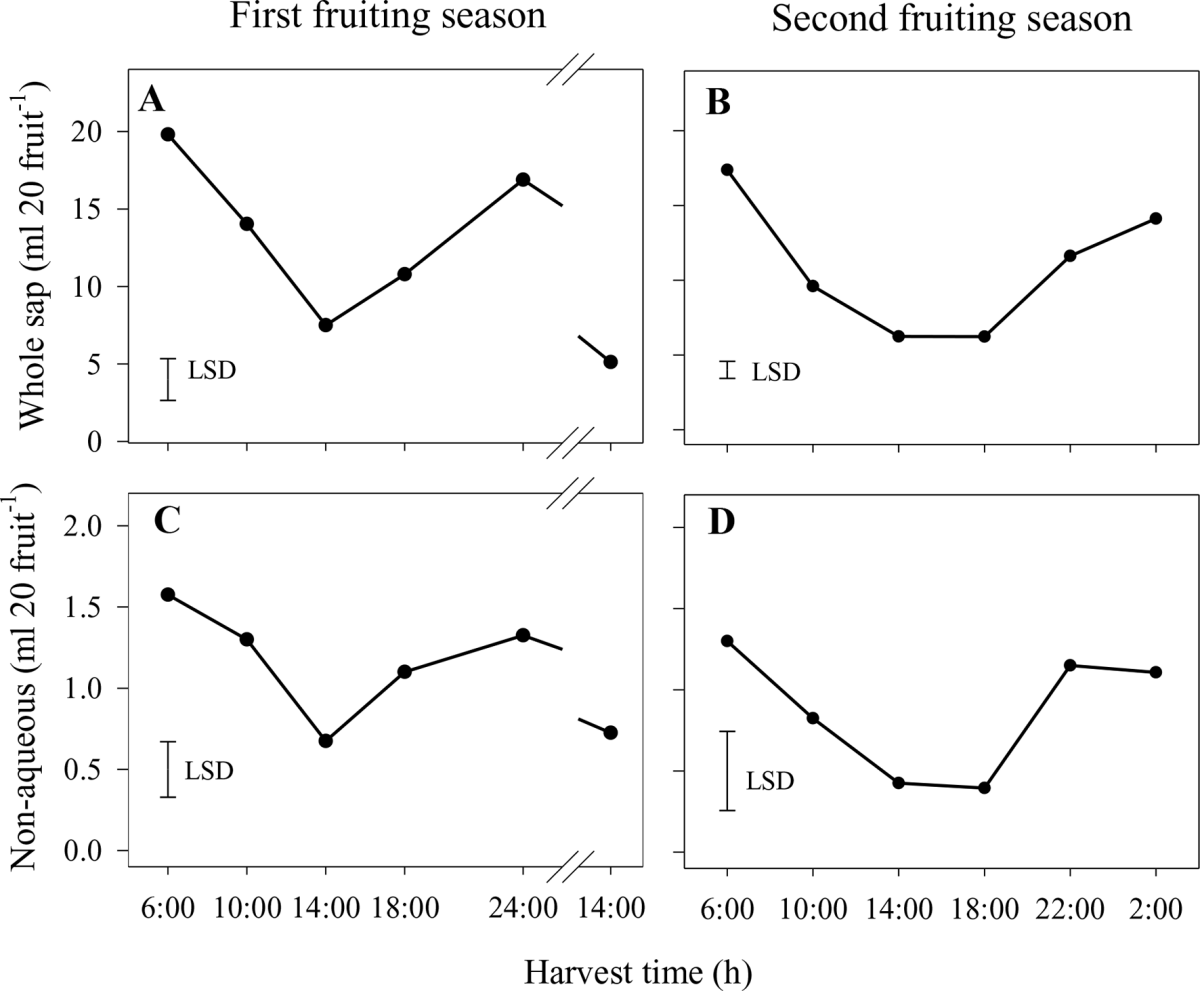
Volumes of whole sap **(A** and **B)** and nonaqueous phase **(C** and **D)** of Honey Gold mango fruit harvested over the diurnal harvest cycle in the first **(A** and **C)** and second **(B** and **D)** consecutive fruiting seasons (Experiment 1). The vertical bar on each graph indicates the LSD value (*P* ≤ 0.05) for comparison of means between harvest times (n = 4). Break lines on the harvest timescales in graphs **(A** and **C)** indicate a gap of 14 h between assessments.

In the first season, the volumes of whole sap and the nonaqueous phase showed similar treatment responses over the diurnal harvest cycle, with the lowest volumes being found for fruit harvested at 14:00 h on both days (**Figures 3A, B**). Similarly, volumes of whole sap and the nonaqueous phase in the second season were lower at 14:00 and 18:00 h than at other harvest times (**Figures 3C, D**). Due to the high proportion of the aqueous phase in the whole sap (viz., > 90% across all harvest times and generally with little variation between harvest times), its volumes over the diurnal harvest cycle followed a pattern nearly identical to that of the whole sap in both seasons (data not shown).

#### Effect of Diurnal Harvest Cycle on Sap Aroma Volatiles

**Figure 4:**
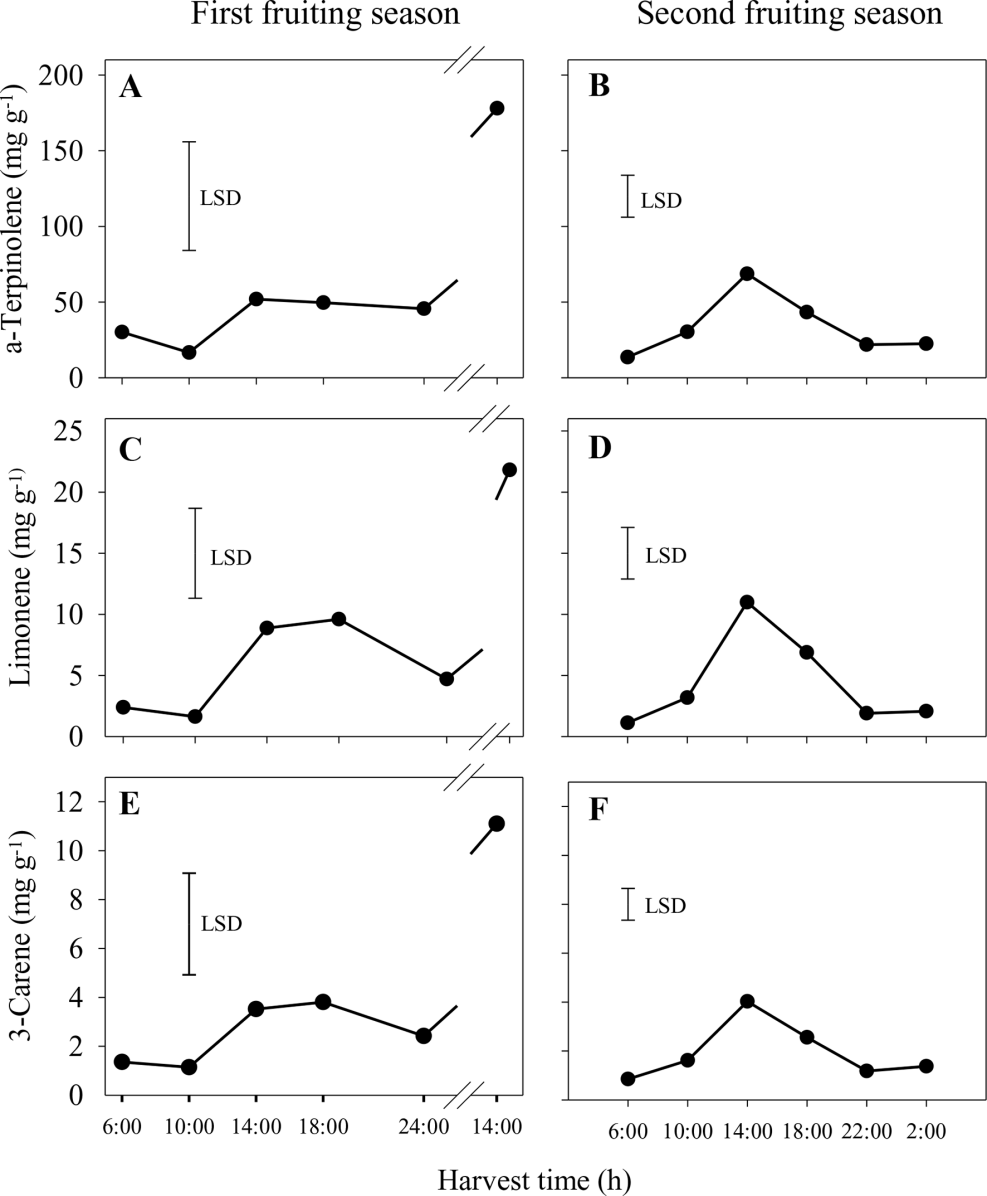
Concentrations of the aroma volatiles α-terpinolene **(A** and **B)**, limonene **(C** and **D)** and 3-carene **(E** and **F)** in the whole sap of ‘Honey Gold’ mango fruit harvested over the diurnal harvest cycle in the first **(A**, **C** and **E)** and the second **(B**, **D** and **F)** consecutive fruiting seasons (Experiment 1). The vertical bar on each graph indicates the LSD value (*P* ≤ 0.05) for comparison of means between harvest times (n = 4). Break lines on the harvest timescales in graphs **(A, C** and **E)** indicate a gap of 14 h between assessments.

In the first season, the concentrations of the main volatiles, α-terpinolene, limonene and 3-carene, were significantly higher in whole sap from fruit harvested at 14:00 h from the second harvest day compared to all other harvest times (**Figures 4A, C, E**). Similar patterns of volatile concentrations in whole sap were observed for 2-carene (ranging from 0.1 to 1.6 mg g^−1^) and *p*-cymene (0.04–0.7 mg g^−1^), whereas differences for α-terpinene (0.4–2.2 mg g^−1^) were not significant (data not shown). In the second season, the concentrations of α-terpinolene, limonene and 3-carene increased significantly from 6:00 h to peak at 14:00 h and then reduced markedly by 22:00 h (**Figures 4B, D, F**). There were similar concentration patterns for 2-carene (0.10–0.56 mg g^−1^), *p*-cymene (0.02–0.11 mg g^−1^) and α-terpinene (0.34–1.44 mg g^−1^) (data not shown). In both seasons and of those volatiles measured, α-terpinolene was that with the highest concentration in whole sap.

**Figure 5:**
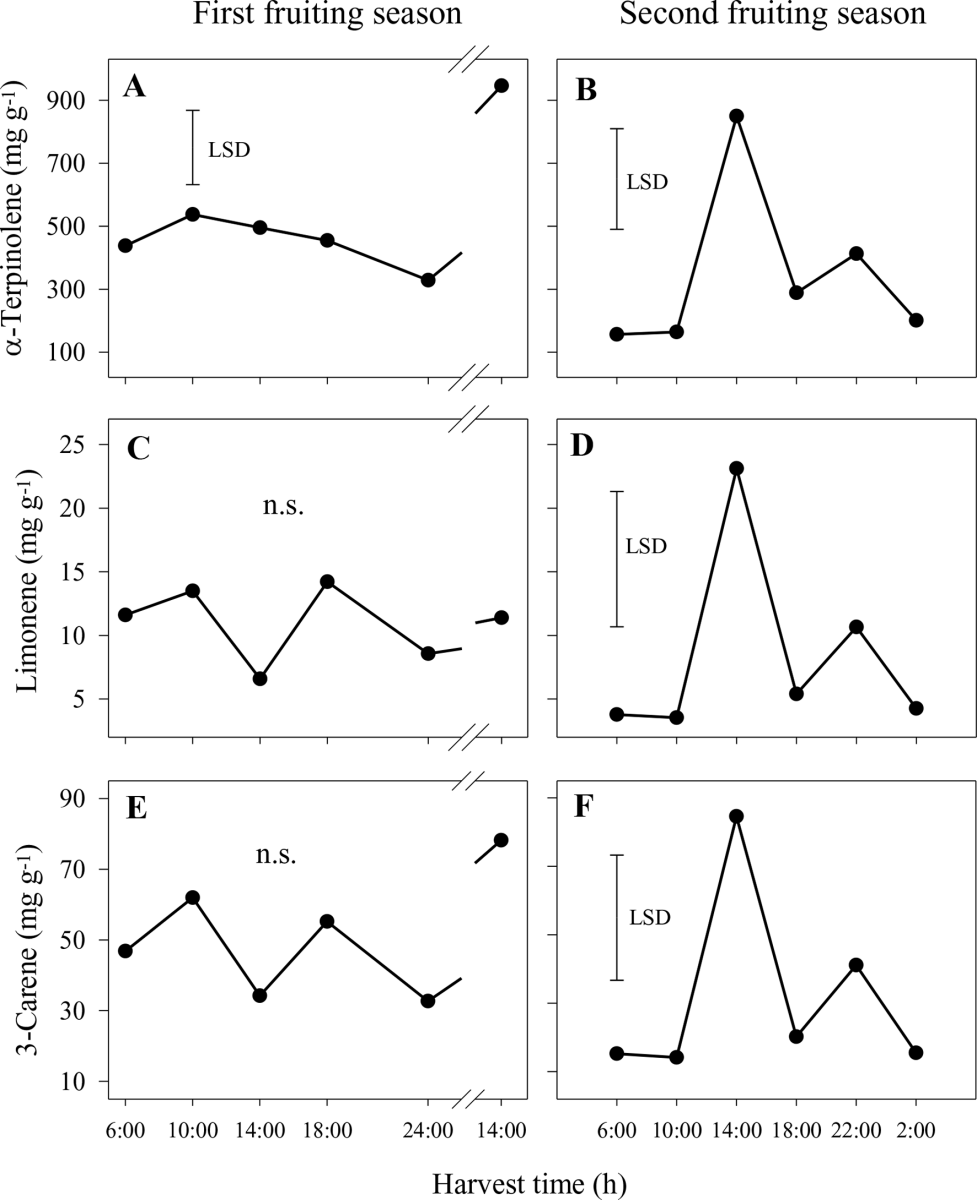
Concentrations of the aroma volatiles α-terpinolene **(A** and **B)**, limonene **(C** and **D)** and 3-carene **(E** and **F)** in the nonaqueous phase of the sap of ‘Honey Gold’ mango fruit harvested over the diurnal harvest cycle in the first **(A**, **C** and **E)** and the second **(B**, **D** and **F)** consecutive fruiting seasons (Experiment 1). The vertical bar on each graph indicates the LSD value (*P* ≤ 0.05) for comparison of means between harvest times (n = 4). n.s. indicates no significant difference. Break lines on the harvest timescales in graphs **(A**, **C** and **E)** indicate a gap of 14 h between assessments.

In the nonaqueous phase in the first season, concentrations of α-terpinolene were significantly higher at 14:00 h on the second day than at other harvest times, whereas differences were not significant for limonene and 3-carene (**Figures 5A, C, E**). A similar pattern of higher concentration at 14:00 h was observed for *p*-cymene (1.2–6.1 mg g^−1^), whereas differences were not significant for 2-carene and α-terpinene (data not shown). In the second season, the concentrations of 2-carene, 3-carene and α-terpinene were also significantly higher at 14:00 h compared to other times (**Figures 5B, D, F**). Similar patterns were observed for 2-carene (2–12 mg g^−1^), *p*-cymene (0.4–2.6 mg g^−1^) and α-terpinene (3–21 mg g^−1^) (data not shown).

In the aqueous phase, concentrations of aroma volatiles were much smaller in both years compared to those in the nonaqueous phase. α-Terpinolene was the predominant compound (0.11–0.81 mg g^−1^), followed by 3-carene (0.014–0.071 mg g^−1^) and α-terpinene (0.003–0.019 mg g^−1^) (data not shown). The concentrations of these three compounds, in addition to the concentrations of 2-carene, *p*-cymene and α-terpinene, were significantly higher in samples collected between 10:00 and 14:00 h than in those from other harvest times in the first season (data not shown). In contrast, there were no significant differences among harvest times in the second season for any of these six volatile compounds (data not shown).

#### Field Temperature and RH

Field temperatures across diurnal harvest times increased similarly in both seasons from around 20°C at 6:00 h to ca. 30°C at 10:00 h and thence to 36 to 39°C at 14:00 h, followed by a decrease to 24 to 27°C at 22:00 to 24:00 h (data not shown). RH was lowest at 37 to 38% at 14:00 h, increasing to 91 to 100% at each of 24:00 and 6:00 h (data not shown).

### Experiment 2: Effect of Sap Components on SB and I-USB

Whole sap and spurt sap from afternoon-harvest fruit and applied to afternoon-harvest fruit resulted in increased severity of SB in 100% of treated fruit compared to either water or ooze sap, which did not result in any SB injury (**Table 1**). Likewise, whole sap and spurt sap from afternoon-harvest fruit and applied to morning-harvest fruit resulted in increased severity of SB in 50 to 57% of treated fruit compared to either water or ooze sap. The spurt sap from morning-harvest fruit resulted in reduced SB severity compared with afternoon-harvest fruit.

**Figure 6:**
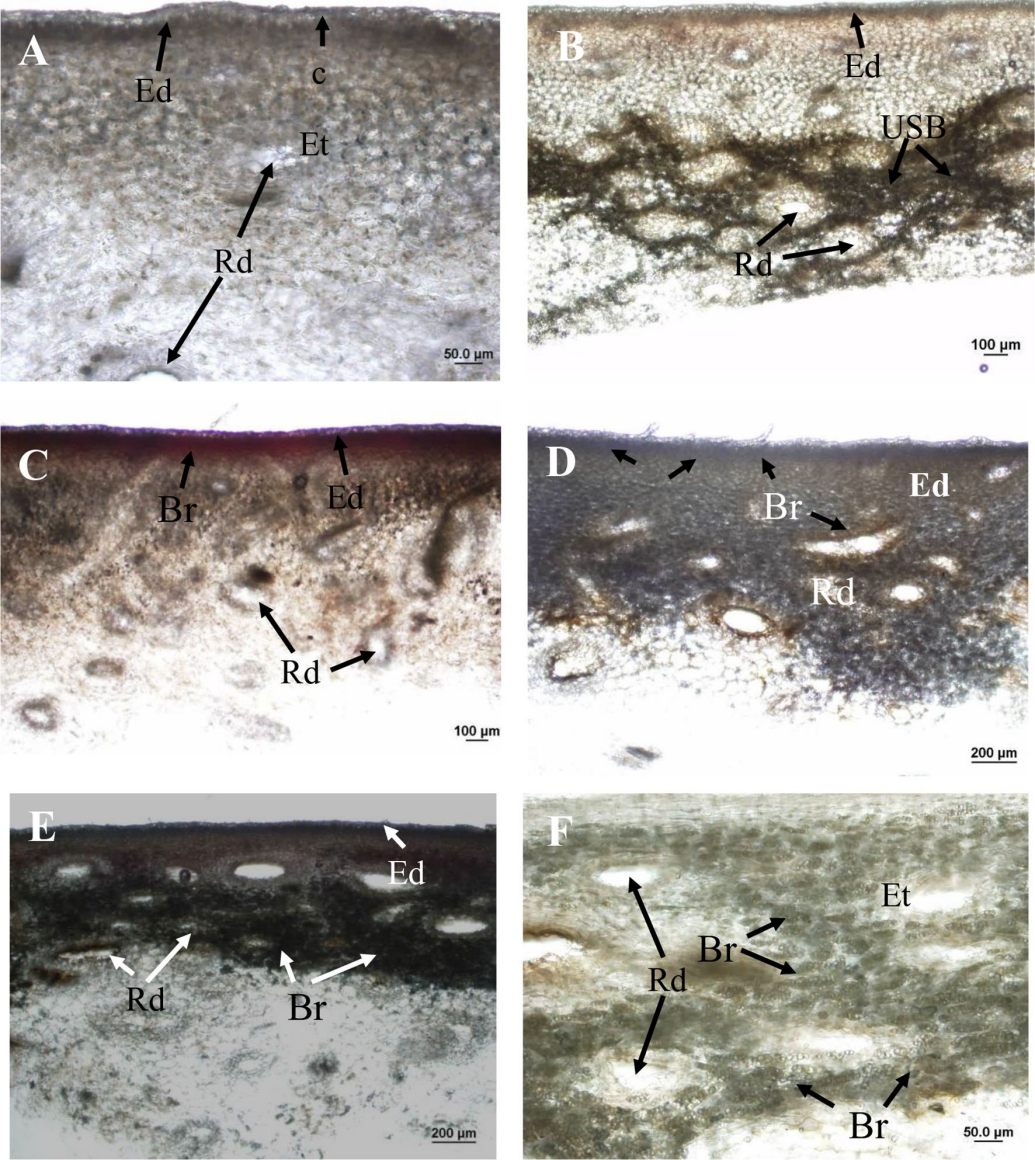
Hand sections of ‘Honey Gold’ mango fruit samples (Experiment 2). **(A)** Control tissue (i.e. distilled water application) showing no browning beneath the epidermis or around resin ducts; **(B)** USB-affected skin tissue showing dark-browning beneath the epidermis and around resin ducts; **(C)** skin browning (SB) due to topical spurt sap application showing mild browning in the epidermis; **(D)** I-USB due to topical terpinolene showing browning beneath the epidermis and around resin ducts; **(E)** I-USB due to topical limonene showing browning beneath the epidermis; and **(F)** I-USB due to topical limonene showing browning around resin ducts. Ct, cuticle; Rd, resin duct; Ed, epidermal cells; Et, epithelial cells; USB, under-skin browning; Br, browning. Scale bars = 20 μm **(B)**, 50 μm **(A** and **F)**, 100 μm **(B** and **C)** and 200 μm **(D** and **E)**.

Applications of the oil fraction components terpinolene and limonene onto fruit resulted in increased severity of induced brown-coloured symptoms (I-USB) to the surrounding skin compared to water or pure 2-carene treatments (**Table 2**). Microscope examinations suggested no apparent difference in severity of I-USB symptoms between terpinolene versus limonene applications. Induced brown-coloured symptoms from terpinolene and limonene application were similar, but not identical, to USB (**Figure 6**). Both of these sap components illustrated had the potential to cause damage.

### Anatomical Observations

Under-skin browning symptoms were localised in subepidermal parenchyma tissue and were not associated with the cuticle (**Figure 6B**). There was no browning of the cuticle through the fruit surface that comprised the waxy cuticle and epidermal and hypodermal cell layers. Under-skin browning–affected tissues showed dark brown parenchyma cells around epithelial cells that line the resin ducts (**Figure 6B**). No browning was observed in healthy fruit tissues treated by topical water application (**Figure 6A**).

Topical sap application caused mild SB symptoms in the epidermis (**Figure 6C**). Compared to USB symptoms, I-USB symptoms caused by the main nonaqueous fraction sap components terpinolene and limonene reflected distinct browning beneath the epidermal layer and around resin ducts (**Figures 6D, E**), as well as similar dark brown parenchyma cells surrounding epithelial cells (**Figures 6B, F**).

## Discussion

A strong association between harvest time during the diurnal cycle and USB incidence and severity was revealed in this study. Contrary to the initial working hypothesis, Honey Gold fruit harvested at night and in the early morning were less sensitive to A-USB as induced by a ‘standardised test’ that comprised light skin abrasion followed by storage at 12 to 14°C for 6 days before fruit ripening and assessment (**Figure 1**). Likewise, USB incidence and severity on nonabraded areas, which likely reflect ‘regular’ USB occurring under commercial conditions, were also lower on fruit harvested at night and in the early morning compared to those harvested in the afternoon (**Figure 2**). Marques et al. (2012) reported that USB often appears around visible physical injury sites on fruit, such as those that can develop during prolonged transport. Overall, diurnal effects on USB both under typical commercial conditions and when using a ‘standardised test’ regime were clearly demonstrated. This suggests that harvesting fruits in the afternoon in combination with physical damage contribute to the loss of cellular integrity that leads to USB.

Based largely on anecdotal evidence, it was hypothesised that the incidence and severity of USB on Honey Gold mango would be at least partly due to sap effects. With ‘Samar Bahisht Chaunsa’ mango fruit, the severity of sapburn injury was higher on harvested at noon and in the afternoon than on those harvested at other times (Amin et al., 2008). The present study confirmed that variation in A-USB and USB incidence and severity of Honey Gold mango fruit across the diurnal harvest cycle was evidently associated with putative differences in sensitivity of the mango fruit skin and/or changes in the composition of potentially phytotoxic sap.

Sap collected from fruit harvested in the afternoon caused more damage when applied to abraded skin than did sap from fruit harvested in the morning, which caused relatively little damage (**Table 1**). These differences suggest the possibility of increasing concentrations of phytotoxic components in the sap of afternoon-harvested fruit. Moreover, it was clear that spurt sap, which contains a high proportion of nonaqueous compounds in Kensington Pride mango (O’Hare et al., 1999), and the nonaqueous sap phase (oil fraction) were responsible for the damage. Thus, afternoon sap, especially afternoon spurt sap, evidently contributed to the relatively greater sensitivity of afternoon-harvested fruit to USB. This conclusion accords with another sapburn study, which showed that skin damage on Kensington Pride mango was associated mainly with the upper nonaqueous phase (Loveys et al., 1992). Maqbool et al. (2007) reported that spurt sap exuded within the first 15 s after removing the pedicel of fruit was most injurious to the skin of ‘Chaunsa’ mango. The current study with Honey Gold mango shows that afternoon spurt sap resulted in less SB when applied to fruit picked in the morning compared to those harvested in the afternoon (**Table 1**). This observation suggests that the skin of afternoon harvested fruit is also more sensitive.

The pattern of higher concentrations of key aroma volatiles compounds in sap from fruit harvested at 14:00 h than in that from those harvested at early morning or night was likely associated with the higher A-USB and USB susceptibility of fruit harvested at 14:00 h. Under high temperatures and low RH, it is probable that afternoon-harvested fruits have lower water content as it has been reported in apples (Link et al., 1998) and avocados (Schroeder and Wieland, 1956). An attendant shrinkage response could concentrate aroma compounds in the sap of mango fruit. It has been established that the apoplasmic water potential gradient is correlated with laticifer and leaf turgor pressures in water-stressed plants (Downton, 1981; Milburn et al., 1990). Moreover, reduced sap flow into tomato fruit is related to water potential gradient between fruit and stem (Johnson et al., 1992). In mango, turgor pressure in resin ducts is maintained because the sap contains high levels of nondialysable and nonstarchy carbohydrate (Saby John et al., 2003). These osmolytic compounds allow high water status to be maintained in laticifers, even under plant water deficit stress (Pongsomboon et al., 1991). It follows that harvesting in the afternoon is likely to increase apparent Honey Gold fruit susceptibility to USB because of relatively higher concentrations of phytotoxic volatile components, including/especially in the nonaqueous phase of the fruit sap (**Figure 5**).

The phytotoxic response of Honey Gold fruit sap to I-USB was related to concentrations of specific sap components. For example, topical application to the fruit skin of terpinolene and limonene, both major components of the oil fraction, resulted in I-USB damage (**Table 2**). In contrast, topical applications of carene and distilled water did not induce I-USB. Although I-USB symptoms caused by terpinolene and limonene were not identical to USB per se (**Figure 6**), similarities clearly indicate the capacity for sap components to cause USB under potentially inductive commercial conditions. Terpinolene and upper phase sap also caused tissue damage in Kensington Pride mangoes, with associated subsequent enzymatic browning (Loveys et al., 1992). Anatomically, both USB (**Figure 6B**) and I-USB symptoms (**Figures 6D–F**) involved dark browning around the epithelial cells that lined resin ducts, whereas sap application resulted in slight browning in the epidermis (**Figure 6C**). Roles for terpinolene and limonene in USB development were also supported by observations that their concentrations can be higher in the afternoon harvest sap relative to morning harvest sap. Further work is warranted to characterise the anatomy of USB versus I-USB symptoms, such as those caused by sap volatiles components, and also into the enzymic biochemistry of the browning process.

In conclusion, harvesting at night or in the early morning increases Honey Gold fruit resistance to USB compared with harvesting in the late morning or afternoon. This impact of the diurnal harvest cycle on mango fruit sensitivity to skin disorders, such as USB, and the association of such impact with specific compositional differences in sap phytotoxicity have not previously been reported. A key contributing factor to more USB expressing in afternoon-harvested fruit is the relative increase in phytotoxic volatile components in their sap. In an applied context, these results have informed a harvest practice change from traditional morning and afternoon harvesting to night and early morning harvesting of Honey Gold mango fruit in Australia aiming to reduce the incidence and severity of USB (Gavin Scurr, personal communication). Added benefits from night and early morning harvesting include less stress on mango pickers because of relatively lower field heat temperatures and reduced ambient fruit temperatures at harvest. Thus, night and early morning harvesting offers opportunity to improve production economics and the fruit quality offered to consumers.

## Data Availability

The datasets generated for this study are available on request to the corresponding author.

## Author Contributions

AS reviewed the literature, conducted the experiments, collected, collated and analysed the data and drafted the manuscript, including figures and tables. PH, DJ and AM contributed to conception and design of the study and, together with JM, provided critical feedback on interpretation of data and discussion of the findings. GL contributed to the conception and conduct of the study. RW and HS advised and contributed to undertaking and interpretation in the areas of microscopy and sap composition, respectively. All authors participated in writing the manuscript and final approval of the present version to be published.

## Funding

This research was funded by Horticulture Innovation Australia Ltd. using voluntary contributions from Piñata Farms matched by funds from the Australian Government. In-kind and financial supports were also provided by the Queensland Department of Agriculture and Fisheries (DAF) and The University of Queensland. We acknowledge the Queensland Alliance for Agriculture and Food Innovation and DAF for the use of specialised laboratory facilities and assistance. AS gratefully acknowledges the receipt of an ACIAR John Allwright Fellowship for financial support of her PhD study in Australia and participation in ‘ACIAR project HORT 2012/098—Improved postharvest management of fruit and vegetables in the Southern Philippines and Australia’. Some of the results of this research were partly submitted as an abstract and presented at the ‘2017 International Tropical Agriculture Conference’, Brisbane, Australia.

## Conflict of Interest Statement

The authors declare that the research was conducted in the absence of any commercial or financial relationships that could potentially be construed as a potential conflict of interest.
